# Supplementary material for: Continuous glucose monitoring in older inpatients with type 2 diabetes and cognitive impairment: an open single-arm feasibility study
Source: BMJ Open. 2026 Sep 11;16(9):e118196. doi: 10.1136/bmjopen-2026-118196 (PMC13583853; doi:10.1136/bmjopen-2026-118196)
Supplement: Supplementary file 1 [file bmjopen-2026-118196.reviewer_comments.pdf]

PEER REVIEW HISTORY

BMJ Open publishes all reviews undertaken for accepted manuscripts. Reviewers are asked to complete a checklist review form and are provided with free text boxes to elaborate on their assessment. These free text comments are reproduced below.

ARTICLE DETAILS

Title (Provisional)

Continuous Glucose Monitoring in Older In-patients with Type 2 Diabetes and Cognitive Impairment -An open single-arm feasibility study

Authors

Donat Ergin, Busra; Mattishent, Katharina; Minihane, Anne Marie; Holt, Richard Ian Gregory; Murphy, Helen; Dhatariya, Ketan; Hornberger, Michael

---

VERSION 1 - REVIEW

Reviewer 1

Name de la Torre, Rafael

Affiliation Hospital del Mar Medical Research Institute, Integrative Pharmacology and Systems Neuroscience Research Group, Neuroscience Research Program

Date 31-Mar-2026

COI None

Ref. bmjopen-2026-118196

Continuous Glucose Monitoring in Older In-patients with Type 2 Diabetes and Cognitive Impairment -An open single arm feasibility study by Donat Ergin and co-workers

The practicality, acceptability, and tolerability of employing a medical device that enables continuous glucose monitoring in elderly patients with type 2 diabetes and cognitive impairment are assessed in this publication. The overall conclusion is that patients with cognitive impairment who might have trouble (as outpatients) monitoring glucose concentrations using point-of-care test (POCT) measurements of capillary blood glucose can use this device for a brief period of time in a feasible, acceptable, and well-tolerated manner. Since T2DM patients frequently use these devices, the results are not surprising. The involvement of patients with cognitive impairment is the main innovative contribution.

There are no comments on the cost effectiveness of using these devices ($1,200 to $3,600) vs. the POCT (1000$) approach on a year basis.

Researchers used the AMT and Mini-ACE tests to assess subjects' cognitive performance. Examining any improvement in cognitive function linked to appropriate glucose control should be one of the study's most interesting findings. The Mini-ACE test appears more sensitive, and after ten days of using the device, a slight improvement might be seen, whereas the AMT test appears unresponsive to any change. When giving these cognitive exams, learning effects may be quite important in this brief time frame. To reduce learning effects, the Mini-ACE has three different variants (A, B, and C). This may affect findings in the cognitive realm and is not discussed in the text.

**Reviewer**	**2**
**Name**	**Lee, Alexandra**
**Affiliation**	**UCSF**
**Date**	**05-Jun-2026**
**COI**	**None**

Overall, this is a simple and well-conducted study evaluating the feasibility and acceptability of continuous glucose monitors in hospitalized older adults with both type 2 diabetes and cognitive impairment. I have a few minor concerns:

1. There may be some data errors in Table 1 – please check that the maximum BMI is 74 and maximum HbA1c is 154mmol/mol (16.2%) are truly correct.

2. For the skin tolerability result in Chart 1, it would be helpful to see the percent with Neutral and negative responses separately (so there are three response categories rather than 2). It seems important to understand how many people have negative impressions of the skin sensor sensation.

3. In the discussion section, it is mentioned that recruitment was restricted to a specific ward of the hospital. This should be included in the methods section, within "setting" or "recruitment process."

4. Given how quickly the CGM field is moving, I would refrain from statements stating "this is the first study…," because there could be a publication tomorrow on this same topic.

VERSION 1 - AUTHOR RESPONSE

Reviewer: 1
Dr. Rafael de la Torre, Hospital del Mar Medical Research Institute, Universitat Pompeu Fabra Facultad de Ciencias de la Salud y de la Vida
Comments to the Author:

The practicality, acceptability, and tolerability of employing a medical device that enables continuous glucose monitoring in elderly patients with type 2 diabetes and cognitive impairment are assessed in this publication. The overall conclusion is that patients with

cognitive impairment who might have trouble (as outpatients) monitoring glucose concentrations using point-of-care test (POCT) measurements of capillary blood glucose can use this device for a brief period of time in a feasible, acceptable, and well-tolerated manner. SThe involvement of patients with cognitive impairment is the main innovative contribution. There are no comments on the cost effectiveness of using these devices ($1,200 to $3,600) vs. the POCT (1000$) approach on a year basis.
Researchers used the AMT and Mini-ACE tests to assess subjects' cognitive performance. Examining any improvement in cognitive function linked to appropriate glucose control should be one of the study's most interesting findings. The Mini-ACE test appears more sensitive, and after ten days of using the device, a slight improvement might be seen, whereas the AMT test appears unresponsive to any change. When giving these cognitive exams, learning effects may be quite important in this brief time frame. To reduce learning effects, the Mini-ACE has three different variants (A, B, and C). This may affect findings in the cognitive realm and is not discussed in the text.

Answer: Thank you for your positive review of our article.

Re: "Since T2DM patients frequently use these devices, the results are not surprising." **This is an interesting comment. In fact, CGM is much less used in T2DM than in T1DM and even less in people with cognitive impairment and T2DM, the population of our research. We would argue, therefore, that the findings are very novel, as they address a highly vulnerable population which is commonly not using CGM.**

Re: "There are no comments on the cost effectiveness of using these devices ($1,200 to $3,600) vs. the POCT (1000$) approach on a year basis."
Thank you for highlighting this. Indeed, we did not conduct a health economics analysis, since this was a feasibility study. We acknowledge that this is a shortcoming and should be addressed in future studies in the discussion chapter; "*Based on that, future research should be considered with larger inpatient populations in the community to evaluate whether CGM use can improve glucose management and clinical outcomes compared to standard care, while also assessing its cost-effectiveness in this setting.*".

Re: "Examining any improvement in cognitive function linked to appropriate glucose control should be one of the study's most interesting findings."
We agree that the cognitive findings are of interest. However, the short follow-up duration (10 days), due to hospital admission, is unlikely to provide meaningful cognitive changes due to glucose control only. Instead, many other factors could have influenced the cognitive changes seen in the results. We have added this point now in the discussion: "*Since short follow-up duration and use of masked CGM are unlikely to provide meaningful cognitive changes, this might have been due to patients having recovered from their conditions, they were admitted for in the first place.*"

Reviewer: 2
Dr. Alexandra Lee, UCSF
Comments to the Author:

Overall, this is a simple and well-conducted study evaluating the feasibility and acceptability of continuous glucose monitors in hospitalized older adults with both type 2 diabetes and cognitive impairment. I have a few minor concerns:

1.There may be some data errors in Table 1 – please check that the maximum BMI is 74 and maximum HbA1c is 154mmol/mol (16.2%) are truly correct.

Answer: Thank you for spotting these extreme values. We have double-checked the values in Table 1 and can confirm that the maximum BMI and maximum HbA1c values are correct.

2. For the skin tolerability result in Chart 1, it would be helpful to see the percent with Neutral and negative responses separately (so there are three response categories rather than 2). It seems important to understand how many people have negative impressions of the skin sensor sensation.

Answer: Thank you for the excellent suggestion. We have updated Chart 1 and show now neutral responses and negative responses separately. Since there was no negative feedback, we added this statement on the table footnote: "*There were no 'No / Negative feedback' answers".*

3. In the discussion section, it is mentioned that recruitment was restricted to a specific ward of the hospital. This should be included in the methods section, within "setting" or "recruitment process."

Answer: Thank you for the suggestion. We have added now more specific information in this regard to 'Recruitment process' section within the Methods: *"We prioritised geriatric wards and wards where patients were expected to have longer hospital stays".*

4. Given how quickly the CGM field is moving, I would refrain from statements stating, "this is the first study…," because there could be a publication tomorrow on this same topic.

Answer: Thank you for pointing this out. We have now removed these statements from the manuscript.
