# Supplementary material for: Continuous glucose monitoring in older inpatients with type 2 diabetes and cognitive impairment: an open single-arm feasibility study
Source: BMJ Open. 2026 Sep 11;16(9):e118196. doi: 10.1136/bmjopen-2026-118196 (PMC13583853; doi:10.1136/bmjopen-2026-118196)
Supplement: Supplementary file 2 [file bmjopen-16-9-s001.docx]

**Appendix**

**Supplemental Material: CGM Acceptability, Feasibility and Tolerability Questionnaire *(IRAS Project ID: 344263 Version: 6.3.6 Date: 03/05/2024)***

***Acceptability***

1. Could you tell me about how you found wearing the sensor?

……………………………………………………………………………………………….

……………………………………………………………………………………………….

2. Did you notice it physically?

- Yes
- No

3. Please rate your pain with an “x” while fitting CGM.

No pain	Highest level of pain

4. Did it affect your day-to-day activities in hospital?

- Yes (……………………………………………………………………………..)
- No

5. Did you feel the device made any difference to your symptoms/experiences of living with diabetes, dementia, co-morbidities, anxiety, & self-care etc?

- Yes (……………………………………………………………………………..)
- No

6. How confident do you feel to wear CGM sensor?

- Not confident at all
- Somewhat unconfident
- Neutral
- Somewhat confident
- Highly confident
- Prefer not to answer

7. Would likely would you be to recommend it to your friends/family?

- Very unlikely
- Somewhat unlikely
- Neutral
- Somewhat likely
- Very likely
- Prefer not to answer

***Feasibility***

8. How easy or difficult to be fitted CGM sensor?

- Very difficult
- Difficult
- Neutral
- Easy
- Very easy
- Prefer not to answer

9. How easy or difficult to keep CGM sensor up to 10 days?

- Very difficult
- Difficult
- Neutral
- Easy
- Very easy
- Prefer not to answer

***Tolerability***

10. How much did the feeling of having the CGM sensor (if any) under the skin encourage or discourage you from using this product?

- Discouraged very much
- Neither discouraged nor encouraged / did not notice the existence
- Encouraged a little
- Encouraged very much
- Prefer not to answer

11. How convenient was it to use CGM sensor?

- Very inconvenient
- Somewhat inconvenient
- Neutral
- Somewhat convenient
- Very convenient
- Prefer not to answer

12. Overall, how much did you like or dislike using the CGM sensor?

- Disliked very much
- Disliked a little
- Neutral
- Liked a little
- Liked very much
- Prefer not to answer

13. If you have any other recommendations or comments on it, please provide them below.

…………………………….…………………………….…………………………….………………

…………………………….…………………………….…………………………….………………

…………………………….…………………………….…………………………….………………
